# Supplementary material for: Synergistic Cellular Responses Conferred by Concurrent Optical and Magnetic Stimulation Are Attenuated by Simultaneous Exposure to Streptomycin: An Antibiotic Dilemma
Source: Bioengineering (Basel). 2024 Jun 21;11(7):637. doi: 10.3390/bioengineering11070637 (PMC11274164; doi:10.3390/bioengineering11070637)
Supplement: Supplementary file 1 [file bioengineering-11-00637-s001.zip › bioengineering-3066640-supplementary.pdf]

A B

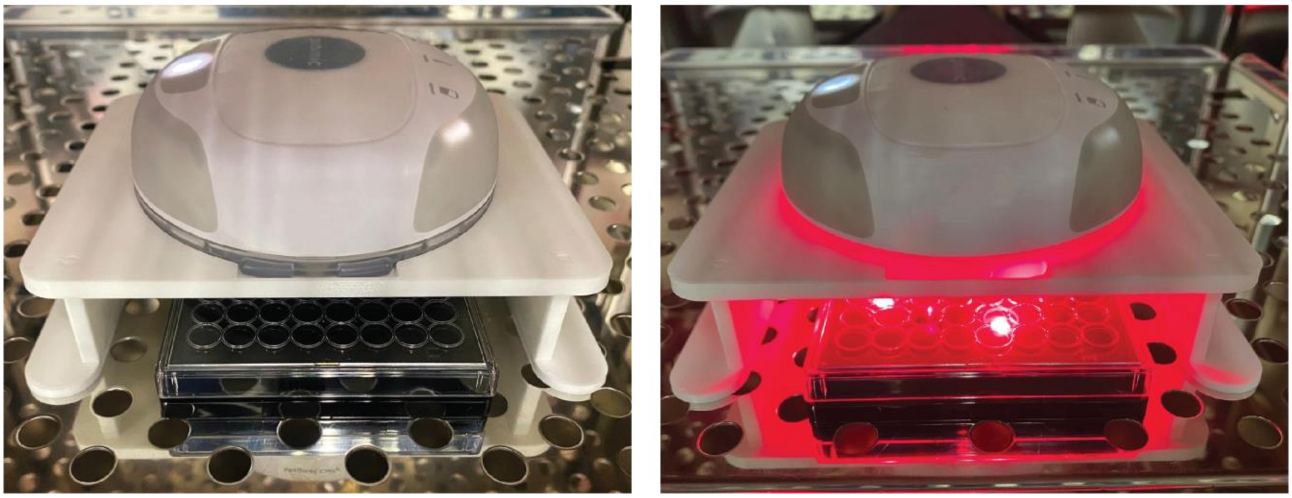

Figure S1. Plated cells subjected to light, magnetic fields, or both in a tissue culture incubator. (A) A typical COMS setup for magnetic fields alone or as a sham; (B) A typical COMS setup for light alone or in combination with magnetic fields.
